# Gigantic hepatic amebic abscess presenting as acute abdomen: a case report

**DOI:** 10.1186/1752-1947-2-325

**Published:** 2008-10-12

**Authors:** TS Papavramidis, K Sapalidis, D Pappas, G Karagianopoulou, A Trikoupi, Ch Souleimanis, ST Papavramidis

**Affiliations:** 1 3rd Department of Surgery, A.H.E.P.A. University Hospital of Thessaloniki, Aristotle's University of Thessaloniki, Thessaloniki, Macedonia, Greece; 2 Department of Pathology, A.H.E.P.A. University Hospital of Thessaloniki, Aristotle's University of Thessaloniki, Thessaloniki, Macedonia, Greece; 3 Department of Anesthesiology, A.H.E.P.A. University Hospital of Thessaloniki, Aristotle's University of Thessaloniki, Thessaloniki, Macedonia, Greece

## Abstract

**Introduction:**

Amebiasis is a parasitic disease caused by *Entamoeba histolytica*. It most commonly results in asymptomatic colonization of the gastrointestinal tract, but some patients develop intestinal invasive or extra-intestinal diseases. Liver abscess is the most common extra-intestinal manifestation. The large number of clinical presentations of amebic liver abscess makes the diagnosis very challenging in non-endemic countries. Late diagnosis of the amebic abscess may lead to perforation and amebic peritonitis, resulting in high mortality rates.

**Case presentation:**

This report describes a 37-year-old white man, suffering from hepatitis B, with a gigantic amebic liver abscess presenting as an acute abdomen due to its rupture. Rapid deterioration of the patient's condition and acute abdomen led to an emergency operation. A large volume of free fluid together with debris was found at the moment of entry into the peritoneal cavity because of a rupture of the hepatic abscess at the position of the segment VIII. Surgical drainage of the hepatic abscess was performed; two wide drains were placed in the remaining hepatic cavities and one on the right hemithorax. The patient was hospitalized in the ICU for 14 days and for another 14 days in our department. The diagnosis of amebic abscess was made by the pathologists who identified *E. histolytica *in the debris.

**Conclusion:**

Acute abdomen due to a ruptured amebic liver abscess is extremely rare in western countries where the parasite is not endemic. Prompt diagnosis and treatment are fundamental to preserving the patient's life since the mortality rates remain extremely high when untreated, even nowadays.

## Introduction

Amebiasis is a widespread parasitic disease caused mainly by *Entamoeba histolytica*. Amebiasis most commonly results in asymptomatic colonization of the gastrointestinal tract, but some patients develop intestinal invasive or extra-intestinal diseases [1]. Of the several extra-intestinal manifestations, liver abscess or hepatic amebiasis is the most common [1]. The large number of clinical presentations of amebic liver abscess (ALA) that have been reported [2] makes the diagnosis, in non-endemic countries, very challenging for the clinician. Late diagnosis of the amebic abscess may lead to perforation in about 2% of ALAs and amebic peritonitis, resulting in high mortality rates [3].

This case is interesting because it reports a ruptured gigantic amebic liver abscess that was surgically treated with success, in a European HBV-positive man who worked as a barman.

## Case presentation

A 37-year-old white man, suffering from hepatitis B, presented to the emergency department with cough, low grade fever and night sweats. He was heterosexual with no history of intravenous drug use and worked as a bartender. Radiological examination of the abdomen and chest revealed no pathologies. Biochemical and hematological profiling showed: SGOT: 71 U/liter, SGPT: 61 U/liter, LDH: 931 U/liter, CRP: 28.33 mg/dl, leucocytosis (12,900/μL) associated with polymorphonucleosis (88.2%), Ht 35% and Hb 11.8 g/dl. The serologic examinations for HIV and hepatitis C were negative, as well as the Mantoux reaction.

**Figure 1:**
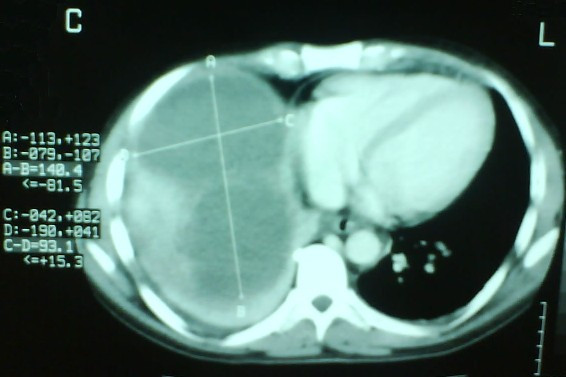
Computed tomography scan with enhancement media showing the hepatic abscess.

**Figure 2:**
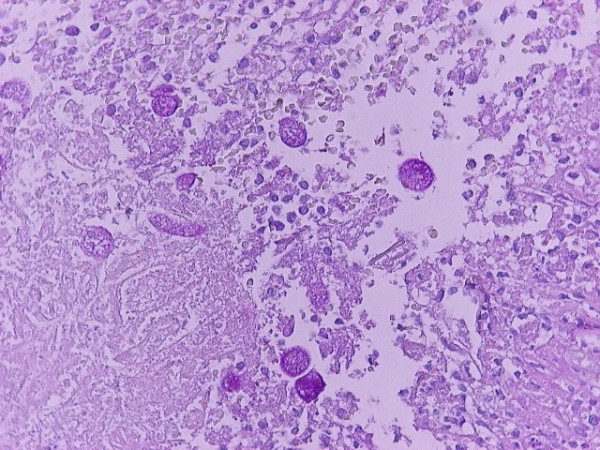
Positive Periodic Acid Schiff staining of *Entamoeba histolytica *(×400).

The next day, the patient presented with dyspnea and auscultation revealed diminished breath sounds with diminished vocal resonance in the right hemithorax. A chest X-ray revealed a pleural effusion in the right hemithorax. Computed tomography (CT) scanning of the chest and abdomen revealed a pleural effusion and a liver abscess (Figure 1). The abscess measured 14 × 9 × 7 cm, occupying a great percentage of the right lobe. An echogram of the liver showed septae within the abscess and for this reason echo- or CT-guided drainage was avoided. An operation was scheduled for the following day, but a rapid deterioration of the patient's clinical condition was observed that evening. The patient was febrile (oral temperature 39.2°C) with hypotension, tachypnea (32 breaths/minute) and tachycardia (110 beats/minute) and signs of an acute abdomen. Therefore, emergency surgery was deemed necessary. During exploratory laparotomy, a large volume of free fluid (~2200 ml) together with debris was found on entry into the peritoneal cavity. A rupture of the hepatic abscess at the position of segment VIII was found <Authors: and surgical drainage of the hepatic abscess (that contained many septae) was performed and two wide drains (32G) were placed in the remaining hepatic cavity. Finally, a thoracic drain tube (Büllau) was placed and gave only yellowish reactive fluid. The patient was hospitalized in the ICU for 14 days and for another 14 days in our department. The cultures of the pus were negative for any microorganisms. The diagnosis of an amebic abscess was made by the pathologists who identified *E. histolytica *in the debris (Figure 2). The patient was discharged receiving metronidazole (Flagyl, Rhone Poulenc Rorer) 500 mg three times a day.

## Discussion

*Entamoeba histolytica *is a protozoan parasite of worldwide distribution. Its general incidence is in areas with tropical and subtropical climates. Various factors such as poor hygiene, diabetes or steroid overuse have been known to predispose to the development of ALA [4]. Chronic consumption of alcohol also seems to predispose to ALA as seen by the fact that most ALA cases occur in people who regularly consume alcohol [4]. Furthermore, immigration and modernization of transport have increased the awareness of ALA even in more developed countries. Additionally, immunosuppression seems to play a role in the development of ALA. This case is interesting because the patient had no travel-related ALA and he lives in a non-endemic country. On the other hand, he had a history of high alcohol consumption that may have played a predisposing role. Also, approximately 10% of patients suffering from ALA have hepatitis, like our patient.

Amebiasis is readily treatable but a delay either in coming to the hospital or in diagnosis can lead to serious complications and even death. Hepatic abscess is the most common non-enteric complication of amebiasis [1]. About 2 to 7% of amebic liver abscesses are complicated by perforation [2,5,6]. Perforation sites mostly include pleuropulmonary structures (72%), the subphrenic space (14%) and the peritoneal cavity (10%) [5]. In our patient, the large hepatic abscess was intact on arrival at the hospital but rupture occurred during hospitalization. Furthermore, surgical exploration revealed that the liver capsule was perforated toward the right subphrenic space. Moreover, as a consequence of downward extension, the hepatic lesion leaked into the peritoneal cavity in the form of a free perforation, causing generalized peritonitis.

Mortality and morbidity of patients with a ruptured ALA are relatively high in comparison to a non-ruptured ALA. Hospitalization averaged 58 days in the report of Meng and Wu [5], while Ken *et al. *reported a mean hospitalization of 14.6 days [3]. Our patient was hospitalized for 14 days in the ICU, and his total hospitalization period lasted for 28 days. Concerning mortality, non-ruptured ALAs have a mortality rate ranging from 4.2 to 4.8% [5,6] when treated with pharmacologic agents, while when untreated, mortality reaches 82% [3]. The mortality of untreated patients is much greater that of treated patients, mostly due to rupture. When the ALAs perforated, the mortality rate reached 23 to 42% [5,7]. When treated immediately with a combination of surgery and a pharmacologic agent (metronidazole), survival improved by 25 to 75% [3].

## Conclusion

Amebic liver abscess is a complication of amebiasis that has to be treated before further complications occur, such as perforations. Acute abdomen due to a ruptured ALA is extremely rare in western countries where the parasite is not endemic. Prompt diagnosis and treatment are fundamental to preserving a patient's life since mortality rates remain extremely high when untreated.

## Consent

Written informed consent was obtained from the patient for publication of this case report and any accompanying images. A copy of the written consent is available for review by the Editor-in-Chief of this journal.

## Competing interests

The authors declare that they have no competing interests.

## Authors' contributions

TSP received the patient in the emergency department, was advising doctor and was involved in drafting the manuscript and revising it critically for content. KS and ChS were auxiliary surgeons and were involved in revising the draft critically for content. DP and GK were pathologists involved in analyzing the specimen and were involved in drafting the manuscript. AT was the main anesthesiologist and was involved in revising the draft critically for content. STP was the main surgeon, carried out strategic planning for treatment of the patient and was involved in revising the draft critically for content. All authors have given final approval of the version to be published.
